# Use of Software as a Medical Device to Improve Therapeutic Adherence in Patients With Hematological Malignancies: Prospective Interventional MargheRITA Study

**DOI:** 10.2196/59662

**Published:** 2026-03-27

**Authors:** Serena Camilla Dalto, Antonella Romanelli, Martina Violati, Carlotta Galeone, Davide Gaudesi, Francesca Sacchi, Massimo Beccaria, Mauro Moroni, Alessandro Ferri, Vittorio Montefusco

**Affiliations:** 1 Hematology Division ASST Santi Paolo e Carlo Milano Italy; 2 Biostatistics & Outcome Research, Statinfo Bicocca Applied Statistics Center (B-ASC) Universita degli Studi di Milano-Bicocca Milano Italy; 3 Advice Pharma Group S.r.l. (Italy) Milano Italy; 4 Oncology Division ASST Santi Paolo e Carlo Milano Italy

**Keywords:** medical device software, digital health, therapy adherence, mobile health, patient outcome, mHealth, medical device, adherence, hematological malignancies, telemedicine, cancer

## Abstract

**Background:**

Hematological malignancies are a global health challenge, with a substantial number of deaths each year. Treatment adherence is crucial for improving patient outcomes in patients with hematological malignancies, but resource limitations and logistical challenges hinder optimal outpatient management. Digital health solutions such as the Remote Intelligence for Therapeutic Adherence (RITA) software as a medical device (SaMD) offer potential solutions by facilitating telemedicine visits and supporting patients in managing their treatment.

**Objective:**

The aim of this study was to evaluate the performance and safety of RITA SaMD in improving patient adherence to treatment protocols for hematological malignancies.

**Methods:**

This prospective clinical investigation enrolled patients with hematological malignancies at Azienda Socio Sanitaria Territoriale Santi Paolo e Carlo in Milan, Italy. The RITA SaMD group used the RITA platform, while the control group comprised historical patients. The primary end point was average therapeutic adherence to the prescribed drug treatment, measured as at least the 80% of the relative dose intensity, at month 3. Secondary end points were based on comparisons between the RITA SaMD group and the control group and included the average therapeutic adherence to the prescribed drug treatment at months 1 and 2, number of emergency room visits for minor and severe complications, and the average hospital stay. Multivariable logistic regression models were used to evaluate the effectiveness of RITA.

**Results:**

Between July and December 2022, 119 patients were included in the analysis (57 in the RITA SaMD group and 62 in the control group). At multivariable analysis, the probability of being adherent to treatment at month 3 in the RITA SaMD group was significantly higher than that in the control group (odds ratio 3.0, 95% CI 1.0-8.8; *P=.*04). A total of 1476 self-reported adverse events (AEs) were collected through RITA SaMD usage, the majority (N=1080) being grade 1 events. During the study visits, 20 AEs were recorded by the study physician (16 in the RITA SaMD group and 4 in the control group). Of the recorded AEs during study visits, 14 were serious AEs (11 in the RITA SaMD group and 3 in the control group). None of the reported AEs was considered related to RITA SaMD usage.

**Conclusions:**

The MargheRITA clinical investigation showed that after 3 months of using RITA SaMD, patients with hematological malignancies had 3 times higher odds ratio of being adherent to the prescribed treatment than the control group. The use of RITA SaMD facilitated the reporting of AEs, reinforcing the role of mobile health apps and software in optimizing patient outcomes. Further research is needed to fully understand its interdisciplinary potential and long-term impact on patient outcomes.

**Trial Registration:**

ClinicalTrials.gov CTI05260203; https://clinicaltrials.gov/study/NCT05260203

## Introduction

Global incident cases of hematologic malignancies have been increasing since 1990, surpassing 1,343,000 in 2019. Still, the age-standardized death rate for all types of hematologic malignancies has been declining [1]. This implies more extended patient management for the health care system.

Treatment adherence is a critical factor in the management of patients with hematological malignancies since poor adherence can result in the emergence of resistance [2,3]. Often, patients underestimate the mild drug- or disease-related symptoms during the treatment, leading to a more severe symptomatology later on that could further impair the patient's adherence to treatment [4]. In addition, many antitumor therapies are now continuous treatments, making therapeutic adherence particularly relevant for patient outcomes. For instance, in a real-world study comparing ibrutinib and acalabrutinib, the two drugs used to treat chronic lymphocytic leukemia, patients were more likely to be adherent to treatment (defined as ≥80% adherence) if they were being treated with ibrutinib, which is administered once daily than if they were being treated with acalabrutinib, which needs to be administered twice daily [5]. Similarly, a study assessing adherence to imatinib treatment in a resource-constrained environment showed that adherence during continuous treatment was related to molecular responses in patients with chronic myeloid leukemia [6]. These data underline the importance of developing innovative approaches to improve therapeutic adherence in patients with hematological malignancies. Therefore, outpatient care models have been developed to offer patients the advantages of home-based treatments, but their implementation and optimization in clinical practice are often challenging as the health care system must deal with resource constraints, exacerbated by factors such as the aging population and improvements in survival rates [1].

In response to these challenges, innovative solutions based on digital health systems such as the use of software as a medical device (SaMD) have recently been designed to complement traditional outpatient care [7]. SaMD can potentially facilitate access to oncological treatments for a wider audience of patients, overcoming geographical and logistical barriers [7].

The onset of the COVID-19 pandemic underscored the urgency of streamlining outpatient management processes [8-10]. Among the most promising strategies to address this need is the development of specific SaMD for oncology, which facilitates telemedicine visits and provides ongoing support for patients in their daily management of the disease, its associated complications, and treatment regimens [8-11]. Compared to nonmedical device software and health care apps, SaMD is developed following the International Organization for Standardization (ISO) 13485 and the European Medical Devices Regulation (EU MDR) 2017/745 (where it is known as medical device software) ensures patient safety and is an essential tool for improving health care quality [12,13].

With this in mind, an SaMD called Remote Intelligence for Therapeutic Adherence (RITA) was developed to serve as a communication platform between doctor and patient and to support the patient in his or her therapeutic journey.

This manuscript briefly describes RITA SaMD and reports the findings of the MargheRITA clinical investigation, which evaluates the performance and safety of the RITA SaMD in improving therapeutic adherence to treatment protocols in patients with hematological malignancies.

## Methods

The CONSORT-EHEALTH (Consolidated Standards of Reporting Trials of Electronic and Mobile Health Applications and Online Telehealth) checklist version 1.6.1 [14] was used to report this study (Multimedia Appendix 1).

### Study Design

This was a premarket, pivotal, confirmatory, prospective, and interventional clinical investigation in patients with hematological malignancies. Patients were screened in a single center in Italy from July to September 2022; the period from the end of enrollment on September 22, 2022, to December 16, 2022, needed to match the RITA SaMD patient to the control group patient.

### Ethical Considerations

This study was conducted under the clinical investigation plan and international guidelines, including the most recent versions of the Declaration of Helsinki, the International Conference on Harmonization guidelines for Good Clinical Practice, EU MDR 2017/745 [13], ISO 14155, as well as all applicable local laws and regulations. The clinical investigation plan was approved by the local ethics committee (Milano Area 1 IEC 0019486; April 21, 2022) and the Italian Ministry of Health (protocol 2028, May 3, 2022). Signed informed consent was obtained from all patients, starting from July 4, 2022 (start of enrollment) to September 22, 2022 (end of enrollment); the period from end of enrollment to December 16, 2022, was used to match the RITA SaMD patients to the control group patients. No compensation was offered to the participants. All data were anonymized to avoid any possible identification and collected in a validated electronic case report form. The clinical investigation is registered at ClinicalTrials.gov (NCT05260203).

### Patient Recruitment

The investigation enrolled individuals who were followed up as outpatients at Azienda Socio Sanitaria Territoriale Santi Paolo e Carlo in Milan who met the following inclusion criteria: comprehension of the investigation's objectives and procedures as well as acceptance to voluntarily sign an informed consent form before any investigation-related activities. In addition, participants needed to be at least 18 years of age and have a diagnosis of a hematological malignancy (encompassing conditions such as symptomatic multiple myeloma, solitary plasmacytoma, amyloidosis, chronic myeloid leukemia, chronic lymphocytic leukemia, lymphocytic lymphoma, Hodgkin lymphoma, B-cell non-Hodgkin lymphoma, T-cell non-Hodgkin lymphoma, acute myeloid leukemia, myelodysplasia, and chronic myeloproliferative disorder). Eligibility also required prior receipt of standard-of-care therapy, irrespective of the administration method, and was open to patients at various therapy stages (in their first or subsequent line of therapy and at the beginning of treatment or not), with a minimum life expectancy of more than 6 months. Exclusion criteria were established to ensure investigation integrity and participant well-being, excluding patients who had solely received radiotherapy, those with clinical conditions hindering treatment adherence, individuals unable to use smartphones or computers, those with major psychopathological conditions or cognitive impairments potentially affecting participation, and patients enrolled in another clinical investigation or in a clinical trial at the time of recruitment and patients with no available information on inclusion/exclusion criteria, pathology, treatments (therapeutic treatments, changes in prescribed regimens, missed doses), and outcomes, thus enabling the evaluation of study end points. Participants had the ability to opt out of the study.

### RITA SaMD

RITA SaMD (Advice Pharma Group S.r.l) was designed and developed as a class I medical device in accordance with ISO 13485 and EU MDR 2017/745 and intended to increase treatment adherence in oncohematological therapeutic regimens.

The RITA SaMD system includes the clinical panel, a mobile app, and the backend. The clinical panel is a web platform managed by the health care professional on which doctors access through a dedicated medical dashboard, after authentication and authorization, to record the patient’s anamnesis and diagnosis, type of drugs, and corresponding posology, both prescribed and effectively taken during follow-up (information verified and recorded at each study visit). The mobile app allows the patient to receive and communicate information about the quality of life and treatment-related adverse events (AEs), providing support in therapy management. The RITA app is designed as a monitoring and support tool for patients with hematological malignancies, allowing clinical professionals to have a clear and complete view of the patient’s state of health. The backend is part of SaMD dedicated to exchanging communications between the RITA mobile app and the clinical panel.

This software is intended to be used by oncohematologists in health care structures (clinical panel module) and by patients in a home care setting (mobile app module). It was designed on the doctor's side as a support for the management of the oncohematological patient and, on the patient's side, as a first aid tool for the management of the most common problems, as well as an efficient and noninvasive means of communication with their doctor. The system also includes the caregiver and general practitioner in the communication group.

RITA SaMD is also designed to communicate and receive information on the quality of life and side effects, thus making the medical doctor aware of the health status of his or her patient population.

**Figure 1:**
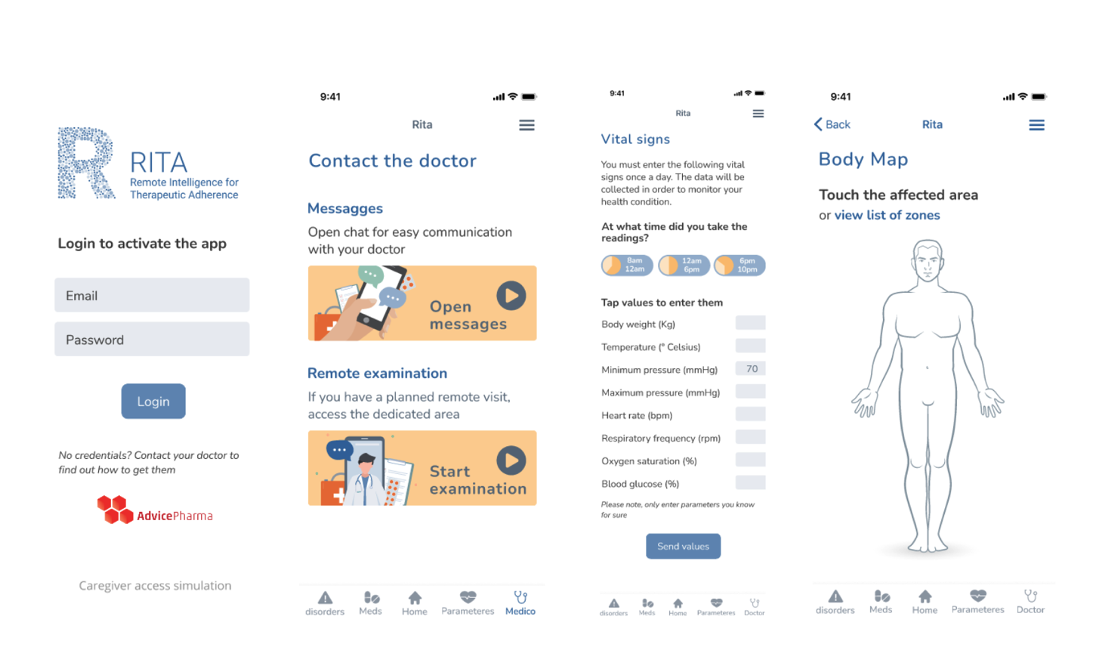
Example screenshots of Remote Intelligence for Therapeutic Adherence (RITA) software as a medical device (SaMD). Physicians were able to access RITA SaMD directly from a computer by using a web-based interface.

Using RITA SaMD, patients could manually notify their daily physical condition as well as disorders, vital parameters (minimum blood pressure, max blood pressure, temperature, heart rate, breath frequency, oxygen saturation, blood sugar, and body weight), and therapy (drug’s name and assumption’s time). RITA SaMD collects patient complaints with a proprietary grading system, which is modeled on Common Terminology Criteria for Adverse Events (version 5.0) [15] (Figure 1). In this way, physicians could manage and resolve mild to moderate symptoms during treatment.

The RITA SaMD database has been developed on an electronic data capture technology (ICE, Advice Pharma), compliant with the regulations for clinical data management to facilitate the study data analysis.

### Study Interventions

The principal investigator granted access to RITA SaMD to each patient prospectively enrolled in the study (RITA SaMD group). The follow-up visits occurred 1, 2, and 3 months after enrollment. The follow-up visits at 3 months coincided with the end of the investigation visit. Each follow-up visit was a routine standard-of-care visit; no investigation-specific assessment was performed. During this visit, the physician paid particular attention to the patient’s reported AE/serious AEs (SAEs). At each follow-up visit, the physician evaluated the self-reported delivered dose intensity.

A control group was also established; this group included patients with hematological malignancies treated in the same hospital in the past 3 years. Each patient in the RITA SaMD group was paired in a 1:1 ratio with a patient in the control group of the same gender, pathology, treatment, and of the closest possible age.

### End Points

The primary end point was the average therapeutic adherence to the prescribed drug treatment, measured as at least 80% of the relative dose intensity, at month 3. Mean treatment adherence was evaluated at the end of months 1, 2, and 3; the primary end point considered the 3-month mean treatment adherence, accounting for the adherence data over 3 months of follow-up. Dose intensity was defined as the ratio of self-reported delivered dose intensity to the prescribed referenced dose intensity (expressed as a percentage) during the investigation period. Data used to calculate the therapeutic adherence were recorded in the clinical panel by the referring physicians; the therapeutic adherence calculation was performed during the analysis. The effectiveness of RITA SaMD use was evaluated by comparing the results of therapeutic adherence with the control group.

At the baseline visit, only for oral medications, routine nursing interventions were provided, as nonoral medications were administered at the clinical site (hospital) by nurses or physicians. Physicians and nurses educated patients on the intake of prescribed therapy in terms of amount and modality, basic nursing care for symptoms, and related complications. Furthermore, for each oral treatment, they verbally instructed patients on how to record information about their prescribed treatments. The physicians asked to document the number of pills assumed according to the assumption scheduling, that is, daily for daily drug assumption, every 7 days for weekly assumption at each visit. No specific questionnaire or standardized tools of recording adherence of oral medications were used, allowing each patient to self-report information as preferred. This mode of recording adherence was used because it is the standard of care in use at the clinical center and because it avoided bias of data collection of this information with respect to matched control group.

During each visit of the study, clinicians reported the therapy administration information reported by patients. The answers were recorded in the electronic case report form. Doses effectively assumed, reason for the dose reductions, percentage of dose reductions, and delays in the administration (both the number of the days and the reason of the delay) were also documented. Only physicians talked with participants during the study visits to gather data. The patients daily tracked the self-reported delivered dose intensity and then reported these data to the physician at study visits at months 1, 2, and 3.

Secondary end points were based on comparisons between the RITA SaMD group and the control group and included the average therapeutic adherence to the prescribed drug treatment at months 1 and 2, number of emergency room (ER) visits for minor and severe complications, and the average hospital stay. Safety secondary end points included physician-reported AEs during the study visits in the RITA SaMD group vs the control group. Other safety secondary end points were only evaluated in the RITA SaMD group and included self-reported AEs and adverse device effects through RITA SaMD usage.

Patient-reported outcomes (PROs) were collected at months 1, 2, and 3 in the RITA SaMD group. Among the questionnaires used were the EQ-5D-5L descriptive system [16], the EuroQol visual analog scale [17], the Instrumental Activities of Daily Living scale [18], and the Activities of Daily Living scale [19].

### Statistical Analysis

The clinical investigation planned a sample size of 112 patients (56 patients in the RITA SaMD group and 56 in the control group) to provide 80% power to detect 20% improvement in treatment adherence after 3 months of RITA SaMD usage, with a 1-sided type I error rate of .05. The enrollment of 124 patients (62 patients per group) was set as the goal to account for a dropout rate of 10%.

The primary analysis included all patients observed until the end of the investigation. The patient demographics were reported using descriptive analyses by tabulating frequencies and percentages for categorical variables and mean and median values, SD, quartiles, and extreme values for continuous variables. Continuous data between RITA SaMD and control groups were compared using a 2-sided Student *t* test, after verifying that the data were normally distributed (based on the Shapiro-Wilk test) and a 2-sided Wilcoxon’s rank-sum test otherwise. For categorical data, comparisons between groups were performed using the chi-square or Fisher exact test, as appropriate.

The effectiveness of RITA SaMD was compared between the RITA SaMD group and the control group by using multivariable logistic regression models, including the following factors (chosen a priori) as the main confounders: age, gender, comorbidities, and disease severity. Adjusted odds ratios (ORs) and the corresponding 95% CIs were estimated and reported. AEs occurring during the investigation follow-up were also described.

The statistical significance limit was accepted as 5%, and the results below this value (*P*<.05) were considered statistically significant. Statistical analyses were performed using SAS system software (v. 9.4; SAS Institute Inc).

## Results

### Patient Characteristics

The first RITA SaMD patient signed the informed consent on July 4, 2022 (start of enrollment), and the last RITA SaMD patient signed the informed consent on September 22, 2022. A total of 62 patients were enrolled in the RITA SaMD group and 62 were, therefore, matched in the control group. Among patients in the RITA SaMD group, 5 did not complete the study: 1 patient was lost to follow-up, 3 patients died from causes not related to the investigation, and 1 patient discontinued treatment after an SAE not related to the investigational device. Thus, 119 patients were analyzed for the primary end point: 57 patients in the RITA SaMD group and 62 in the control group. Baseline patient characteristics are reported in Table 1.

**Table 1:** Baseline patient characteristics.

		RITA^a^ SaMD^b^ group (n=62)	Control group (n=62)	*P* value	
**Gender, n (%)**					.21
	Male, n (%)	37 (59.7)	30 (48.4)		
	Female, n (%)	25 (40.3)	32 (51.6)		
Age (y), mean (SD)		69.3 (15.1)	73.57 (9.9)	.16	
**Pathology, n (%)**					.99
	Monoclonal gammopathy	22 (35.5)	20 (32.3)		
	Chronic myeloid leukemia	4 (6.5)	22 (35.5)		
	Chronic lymphocytic leukemia/lymphocytic lymphoma	9 (14.5)	9 (14.5)		
	Hodgkin lymphoma	3 (4.8)	3 (4.8)		
	B-cell non-Hodgkin lymphoma	12 (19.4)	13 (21)		
	T-cell non-Hodgkin lymphoma	3 (4.8)	3 (4.8)		
	Acute myeloid leukemia	2 (3.2)	2 (3.2)		
	Myelodysplasia	3 (4.8)	4 (4.5)		
	Chronic myeloproliferative syndrome	4 (4.5)	4 (4.5)		
**Treatment phase, n (%)**					.005
	1st line	52 (83.8)	38 (61.3)		
	2nd line or more	10 (16.1)	24 (38.7)		
**Method of treatment administration, n (%)**					.37
	Intravenous	35 (56.5)	30 (51.6)		
	Oral/other	27 (43.5)	32 (48.4)		

^a^RITA: Remote Intelligence for Therapeutic Adherence.

^b^SaMD: software as a medical device.

### Performance

#### Primary End Point

In the primary end point analysis, 4 patients in the RITA SaMD group had missing data at the follow-up study visits. Therefore, the analyses included 115 patients at month 3. At univariable analysis, the proportion of treatment-adherent patients, that is, ≥80% adherent to treatment, in the RITA SaMD group tended to be slightly higher than that in the control group at all time points (46/57, 82.1% vs 50/62, 80.6% at month 1, 43/57, 81.1% vs 45/62, 76.3% at month 2, and 46/57, 85.2% vs 47/62, 77% at month 3) (Table 2).

**Table 2:** Treatment adherence to therapy.

	RITA^a^ SaMD^b^ group (n=57), n (%)	Control group (n=62), n (%)	*P* value
Month 1	46 (82.1)	50 (80.6)	.84
Month 2	43 (81.1)	45 (76.3)	.53
Month 3	46 (85.2)	47 (77)	.27

^a^RITA: Remote Intelligence for Therapeutic Adherence.

^b^SaMD: software as a medical device.

n terms of treatment adherence, univariable analyses revealed that differences between the two groups were not statistically significant, even when considering the mode of treatment administration (intravenous vs oral): treatment adherence was higher in the RITA SaMD Group than in the Control Group [intravenous: 22/3, 71% vs 22/30, 73.3% at Month 1 (*P*=.84), 20/29, 69% vs 16/27, 59.2% at month 2 (*P*=.45), and 23/27, 85.1% vs 18/29, 62% at month 3 (*P*=.09); oral: 24/25, 96% vs 28/32, 87.5% at month 1 (*P*=.26), 23/24, 95.8% vs 29/32, 90.6% at month 2 (*P*=.45), and 23/26, 88.5% vs 29/32, 90.6% at month 3 (*P*=.79)].

A multivariable analysis, adjusted for potential confounders (gender, age, number of concomitant pathologies, and treatment line), was performed. At month 3, the OR of the RITA SaMD group to be adherent to treatment was 3 times higher than that of the control group (adjusted OR 3.0, 95% CI 1.0-8.8; *P*=.04) (Table 3).

**Table 3:** Multivariable logistic regression analysis on the risk of being adherent to therapy over 3 months.

	Adjusted odds ratio (95% CI)	*P* value
Use of RITA^a^ SaMD^b^ app at month 3 (yes vs no)	3.0 (1.0-8.8)	.04
Sex (female vs male)	1.4 (0.5-3.8)	.53
Age (60-79 y vs 60 y)	2.5 (0.6-10.6)	.23
Age (≥80 y vs 60 y)	2.2 (0.5-10.3)	.30
Number of concomitant pathologies (1-2 vs 0)	0.9 (0.1-5.3)	.88
Number of concomitant pathologies (≥3 vs 0)	0.5 (0.1-2.3)	.35
Number of treatment lines (≥1 vs 1)	6.8 (1.4-33.3)	.02

^a^RITA: Remote Intelligence for Therapeutic Adherence.

^b^SaMD: software as a medical device.

#### Secondary End Points

Treatment adherence was analyzed in 118 patients at month 1 and 112 patients at month 2. The multivariable analysis with adjustment for potential confounders showed that the differences between the two groups in terms of treatment adherence were not statistically significant in the first 2 months, with an OR of 1.7 (95% CI 0.61-4.9) at month 1 and an OR of 2.0 (95% CI 0.8-5.6) at month 2.

Regarding ER visits, there were no visits in either group for minor complications. At the end of the study period, there were 9 ER visits in the RITA SaMD group requiring hospitalization and 3 in the control group, totaling 12 ER visits for severe complications. The average hospital stay was 25.3 (SD 22.7) days in the RITA SaMD group and 8.3 (SD 2.1) days in the control group. Due to the small number of events, no statistical test was performed for these end points.

#### Safety (Secondary End Points)

In the RITA SaMD Group, there were 16 AEs reported by the physicians during the study visits (Table 4), most of them grade 2, while in the control group, there were 4 AEs reported (Table 5). During the study, 14 SAEs occurred, 11 of which were in the RITA SaMD group. None of the recorded AEs was related to RITA SaMD usage; therefore, there were no adverse device effects.

**Table 4:** Adverse events recorded by the physician during the study visits—RITAa SaMDb group (n=16).

Body system organ class			Grade 1 (n=1), n (%)		Grade 2 (n=8), n (%)		Grade 3 (n=3), n (%)		Grade 4 (n=2), n (%)		Grade 5 (n=2), n (%)
**Cardiac disorders (n=4)**			0 (0)		3 (75)		1 (25)		0 (0)		0 (0)
	Cardiac failure congestive (n=1)	0 (0)		0 (0)		1 (100)		0 (0)		0 (0)	
	Cardiac failure (n=1)	0 (0)		1 (100)		0 (0)		0 (0)		0 (0)	
	Atrial fibrillation (n=2)	0 (0)		2 (100)		0 (0)		0 (0)		0 (0)	
**General disorders and administration site conditions (n=5)**			0 (0)		3 (60)		0 (0)		0 (0)		2 (40)
	Asthenia (n=2)	0 (0)		2 (100)		0 (0)		0 (0)		0 (0)	
	Disease progression (n=2)	0 (0)		0 (0)		0 (0)		0 (0)		2 (100)	
	Pyrexia (fever) (n=1)	0 (0)		1 (100)		0 (0)		0 (0)		0 (0)	
**Infections and infestations (n=4)**			0 (0)		2 (50)		1 (25)		1 (25)		0 (0)
	COVID-19 infection (n=2)	0 (0)		1 (50)		1 (50)		0 (0)		0 (0)	
	Infection (not specified) (n=1)	0 (0)		1 (100)		0 (0)		0 (0)		0 (0)	
	Septic shock (n=1)	0 (0)		0 (0)		0 (0)		1 (100)		0 (0)	
**Respiratory, thoracic, and mediastinal disorders (n=1)**			0 (0)		0 (0)		1 (100)		0 (0)		0 (0)
	Interstitial lung disease (interstitial pneumonia) (n=1)	0 (0)		0 (0)		1 (100)		0 (0)		0 (0)	
**Vascular disorders (n=2)**			1 (50)		0 (0)		0 (0)		1 (50)		0 (0)
	Hypotensive syncope (anemia lipothymia) (n=1)	1 (100)		0 (0)		0 (0)		0 (0)		0 (0)	
	Cardiovascular disorder (recurrence of circulatory decompensation) (n=1)	0 (0)		0 (0)		0 (0)		1 (100)		0 (0)	

^a^RITA: Remote Intelligence for Therapeutic Adherence.

^b^SaMD: software as a medical device.

**Table 5:** Adverse events recorded by the physician during the study visits—control group (n=4).

Body system organ class		Grade 1 (n=0), n (%)		Grade 2 (n=1), n (%)	Grade 3 (n=3), n (%)		Grade 4 (n=0), n (%)		Grade 5 (n=0), n (%)
**Blood and lymphatic system disorders (n=1)**		0 (0)		0 (0)	1 (100)		0 (0)		0 (0)
	Neutropenia (n=1)	0 (0)	0 (0)		1 (100)	0 (0)		0 (0)	
**General disorders and administration site conditions (n=1)**		0 (0)		1 (100)	0 (0)		0 (0)		0 (0)
	Asthenia (n=1)	0 (0)	1 (100)		0 (0)	0 (0)		0 (0)	
**Hepatobiliary disorders (n=1)**		0 (0)		0 (0)	1 (100)		0 (0)		0 (0)
	Hyperbilirubinemia (n=1)	0 (0)	0 (0)		1 (100)	0 (0)		0 (0)	
**Respiratory, thoracic, and mediastinal disorders (n=1)**		0 (0)		0 (0)	1 (100)		0 (0)		0 (0)
	Dyspnea (persistent fever and difficulty breathing) (n=1)	0 (0)	0 (0)		1 (100)	0 (0)		0 (0)	

Regarding self-reported AEs, a total of 1476 AEs were recorded through RITA SaMD usage (Table 6). There were 1080 minor (grade 1) events, with tiredness being the most frequently reported grade 1 event (n=720).

**Table 6:** Self-reported adverse events in RITAa SaMDb group.c

Body system organ class			Grade 1 (n=1080), n (%)		Grade 2 (n=370), n (%)		Grade 3 (n=26), n (%)
**Cardiac disorders (n=5)**			3 (60)		2 (40)		0 (0)
	Palpitations (n=5)	3 (60)		2 (40)		0 (0)	
**Eye disorders (n=99)**			0 (0)		90 (90.9)		9 (9.1)
	Vision decreased (decrease of vision) (n=99)	0 (0)		90 (90.9)		9 (9.1)	
**Gastrointestinal disorders (n=50)**			41 (82)		7 (14)		2 (4)
	Diarrhea (n=32)	27 (84.4)		4 (12.5)		1 (3.1)	
	Constipation (n=10)	7 (70)		3 (30)		0 (0)	
	Stomatitis (oral cavity mucositis) (n=6)	6 (100)		0 (0)		0 (0)	
	Dyspepsia (difficult digestion) (n=1)	0 (0)		0 (0)		1 (100)	
	Gastric dilatation (stomach distension) (n=1)	1 (100)		0 (0)		0 (0)	
**General disorders and administration site conditions (n=955)**			773 (80.9)		172 (18)		10 (1.1)
	Fatigue (tiredness) (n=858)	720 (83.9)		129 (15)		9 (1.1)	
	Peripheral swelling (swelling in the legs) (n=42)	12 (28.6)		30 (71.4)		0 (0)	
	Pain (ache) (n=30)	17 (56.7)		12 (40)		1 (3.3)	
	Pyrexia (fever) (n=25)	24 (96)		1 (4)		0 (0)	
**Infections and infestations (n=41)**			36 (87.8)		5 (12.2)		0 (0)
	Flu symptoms (n=39)	35 (89.7)		4 (10.3)		0 (0)	
	Conjunctivitis (n=2)	1 (50)		1 (50)		0 (0)	
**Musculoskeletal and connective tissue disorders (n=3)**			3 (100)		0 (0)		0 (0)
	Gait disturbance (walking disorders) (n=3)	3 (100)		0 (0)		0 (0)	
**Nervous system disorders (n=103)**			44 (42.7)		59 (57.3)		0 (0)
	Insomnia (n=55)	6 (10.9)		49 (89.1)		0 (0)	
	Anxiety (n=29)	21 (72.4)		8 (27.6)		0 (0)	
	Taste disorder (n=14)	13 (92.9)		1 (7.1)		0 (0)	
	Tremors (n=3)	3 (100)		0 (0)		0 (0)	
	Cognitive disorder (n=2)	1 (50)		1 (50)		0 (0)	
**Psychiatric disorders (n=14)**			13 (92.9)		1 (7.1)		0 (0)
	Nervousness (n=14)	13 (92.9)		1 (7.1)		0 (0)	
**Renal and urinary disorders (n=14)**			14 (100)		0 (0)		0 (0)
	Pollakiuria (urinate often) (n=13)	13 (100)		0 (0)		0 (0)	
	Urinary incontinence (n=1)	1 (100)		0 (0)		0 (0)	
**Respiratory, thoracic, and mediastinal disorders (n=38)**			17 (44.7)		17 (44.7)		4 (10.5)
	Cough (n=36)	16 (44.4)		17 (47.2)		3 (8.3)	
	Dyspnea (difficulty breathing) (n=1)	0 (0)		0 (0)		1 (100)	
	Hiccups (n=1)	1 (100)		0 (0)		0 (0)	
**Skin and subcutaneous tissue disorders (n=146)**			129 (88.4)		16 (11)		1 (0.7)
	Pruritus (itching) (n=122)	110 (90.2)		11 (9)		1 (0.8)	
	Paresthesia (tingling feeling) (n=21)	17 (81)		4 (19)		0 (0)	
	Skin alterations (n=3)	2 (66.7)		1 (33.3)		0 (0)	
**Vascular disorders (n=8)**			7 (87.5)		1 (12.5)		0 (0)
	Epistaxis – nosebleed (n=8)	7 (87.5)		1 (12.5)		0 (0)	

^a^RITA: Remote Intelligence for Therapeutic Adherence.

^b^SaMD: software as a medical device.

^c^Percentages are based on the total number of cases for each row.

### PROs in Questionnaires

In the RITA SaMD group, 1 patient had complete questionnaires at all time points and 17 patients had complete questionnaires at month 1 and month 2. For each questionnaire, some patients were not considered in the analysis because their assessments were done out of the specified time frame, they reported incorrect values, or data were missing (Table 7).

**Table 7:** Patient-reported outcomes.

		No change, n	Improved, n	Worsened, n
**EQ-5D-5L**				
	Mobility item	6	2	3
	Self-care item	9	1	1
	Usual activities item	9	1	1
	Pain/discomfort	2	3	6
	Anxiety/depression	4	1	6
**Other**				
	EuroQol Visual Analog Scale	2	5	3
	Instrumental Activities of Daily Living scale	—^a^	1	2
	Activities of Daily Living scale	8	—	1

^a^Not applicable.

## Discussion

### Principal Findings

In this clinical investigation of patients with hematological malignancies, patients using the RITA SaMD device had a 3-fold higher risk of being adherent to treatment compared with nonusers after 3 months of use, with patients who received more than 1 line of therapy having a higher likelihood to be adherent. A high number of self-reported AEs of grade 1 was collected in the RITA SaMD group.

Given that patients with hematological malignancies often must adhere to strenuous medication regimens, manage symptoms associated with treatment, and, eventually, experience late effects associated with survivorship, they would benefit greatly from digital technology aimed at decreasing these burdens [20]. Indeed, previous research in the area of mobile health has shown that the use of information and communication platforms can increase the patient’s sense of autonomy and facilitate disease management, resulting in higher quality of life and treatment adherence [21].

The results of a pragmatic pilot trial comparing the use of the digital solution Safety and Adherence to Medications and Self-Care Advice in Oncology (SAMSON) with usual care in patients with hematological malignancies who started oral cancer medicines showed that the use of SAMSON was acceptable, feasible, and helpful in improving medication adherence [22].

Another experience with an online eHealth system for patients to self-report symptoms during cancer treatment, the Electronic Patient Self-Reporting of Adverse-events: Patient Information and Advice (eRAPID) demonstrated how real-time monitoring with PROs ameliorated physical well-being in terms of symptom control measured using the Functional Assessment of Cancer Therapy Scale General Physical Well-Being subscale at 6 weeks. Clinically meaningful benefits in patients’ physical well-being were observed during the first 6 and 12 weeks of treatment when it is expected to see challenges controlling side effects; symptoms stabilized by week 18, when patients reach a steady state of supportive medications or chemotherapy dose adjustments [23]. Based on these observations, the predefined period of observations of 3 months that we adopted in our study was appropriate.

In our study, the number of previous treatment lines affected the odds of being adherent to treatment, with patients who underwent more than 1 line of treatment having a much higher likelihood of adhering to the prescribed drug treatment. This is somewhat in contradiction to what was found by Seal et al [24], who described how adherence declines with increasing lines of treatment but only for oral medications and not intravenous ones. Given that the number of available oral formulations of both chemotherapy and biological therapies is rising and that patients tend to prefer oral therapy, it is worth further exploring the role of mobile health and SaMD in the factors influencing adherence, such as forgetfulness or loss of motivation [25,26].

A total of 1476 AEs were self-reported in the RITA SaMD group with SaMD usage occurrence; the majority of them (73.2%) was of grade 1, showing that app usage facilitated collection of such events and may improve the recording of low-grade AEs compared with traditional methods (namely, AE recording only during medical visits). During the study visits, 20 AEs were recorded by the physician: 16 in the RITA SaMD group and 4 in the control group. Of the collected AEs during the study visits, 14 were considered SAEs (11 in the RITA SaMD group and 3 in the control group). None of the collected AEs was considered to be related to RITA SaMD usage. In a recent study evaluating the factors impacting medication adherence in patients with hematologic malignancies who have undergone allogeneic hematopoietic stem cell transplants, although AEs associated with medication did not directly impact treatment adherence, they increased the patients’ psychological distress [27]. Interestingly, the same study reported that caregiver and clinician support as well as tools to aid medication management were factors that facilitated treatment adherence [27]. RITA SaMD can, therefore, help alleviate stress by providing a way for patients to report their ailments and feel they have clinician support.

RITA SaMD allowed the collection of PROs, including quality of life data. Although the collected data were insufficient to analyze differences in therapeutic adherence relative to self-reported changes in the questionnaires’ items, the fact that RITA SaMD enables the collection of PROs is an added value and may be further explored in the future. Unrecorded PROs are still sadly familiar in the literature; any SaMD that strives to help both patients and physicians work better with the health care system cannot overlook PRO recording and the real-world data it generates [17,28].

Previous research has identified necessary features in mobile health apps and software, such as symptoms and medication tracking [29]. Patients themselves also state that features that facilitate information exchange with their clinicians are important [30]. Our results in patients with hematological malignancies illustrate how RITA SaMD can help any oncology setting to promote patients’ therapeutic adherence while limiting health care resources used in patient management and contribute to optimal patient care.

### Strengths and Limitations

Our clinical investigation has some limitations. This study was undertaken in a single center, which might limit the generalizability of the results. Another limitation is related to the lack of randomization and the unblinded nature of the study, potentially resulting in some biases such as selection bias, recall bias, and bias due to missing data, which were addressed using appropriate methodological approaches during the study design. To overcome the selection bias, the controls originated from the same population used to enroll patients in the intervention group: they had been treated in the same hospital and followed the same treatment protocols in the 3 years before the start of enrollment of patients in the intervention arm. In addition, control patients were identified based on the availability in the hospital’s database of information on inclusion/exclusion criteria, pathology, treatments, and outcomes, thus enabling the evaluation of study end points. Finally, control patients were matched to patients of the intervention group based on the same characteristics, potentially influencing the primary outcome of the study.

As a further limitation, we acknowledge that no specific questionnaire or standardized tools of recording adherence of oral medications were used, allowing each patient to self-report information as preferred. However, this mode was used because it is the standard of care in use at the clinical center and because it avoided bias of data collection of this information with respect to the matched control group. Participants were instructed to report on all the prescribed oncological therapies; however, we could not ensure that their reporting was correct and unbiased, as no methods were in place to verify participants’ self-reporting. Data cleaning verified only if the number of taken doses did not exceed the number of prescribed doses. No imputation was performed; the investigators recorded the available quantities of the drugs effectively assumed in the electronic case report form.

The short follow-up time precludes any conclusion regarding long-term adherence to treatment, despite the promising results. However, the patients considered in the study had different stages of progress in cancer therapy (patients in first-line or later lines, with therapy to be started and with therapies already started); this different composition of the sample implies that the included patients might have different “levels of therapeutic fatigue” and certainly included patients with longer exposure to cancer treatments. The high level of differentiation of the patients included in the study increased the generalization of the results of this study.

Our study also has important strengths. To our knowledge, this is the first clinical investigation showing the effectiveness of an SaMD application in improving therapeutic adherence in patients with hematological malignancies. High-quality methods were implemented, including prospective registration in a publicly available clinical trial database and the use of a control group.

### Conclusion

The MargheRITA clinical investigation revealed that after 3 months of using RITA SaMD, patients with hematological malignancies were 3 times more likely to be adherent to the prescribed treatment than the control group. The use of RITA SaMD facilitated the reporting of AEs and (to a minor extent) of PROs, reinforcing the role of mobile health apps and software in optimizing patient outcomes. This initial clinical investigation has confirmed the performance and safety of RITA SaMD; future studies are needed to prove its interdisciplinary application.

## Appendix

Multimedia Appendix 1 CONSORT-EHEALTH checklist.

## Abbreviations

AE: adverse event
CONSORT-EHEALTH: Consolidated Standards of Reporting Trials of Electronic and Mobile Health Applications and Online Telehealth
eRAPID: Electronic Patient Self-Reporting of Adverse-events: Patient Information and Advice
EU MDR: European Medical Devices Regulation
ISO: International Organization for Standardization
OR: odds ratio
PRO: patient-reported outcome
RITA: Remote Intelligence for Therapeutic Adherence
SAE: serious adverse event
SaMD: software as a medical device
SAMSON: Safety and Adherence to Medications and Self-Care Advice in Oncology
